# Therapeutic outcomes and analysis of Doppler findings in 25 patients with non-ischemic priapism

**DOI:** 10.1038/s41443-023-00719-z

**Published:** 2023-06-13

**Authors:** Conrad von Stempel, Rohaan Shahzad, Miles Walkden, Fabio Castiglione, Asif Muneer, David Ralph, Alex Kirkham

**Affiliations:** 1 Department of Radiology, University College London Hospitals, London, UK; 2 Division of Surgery and Interventional Science, University College London, London, UK; 3 Department of Urology, King’s College Hospital NHS Foundation Trust, London, UK; 4 Department of Urology, University College London Hospitals, London, UK

**Keywords:** Erectile dysfunction, Diagnosis

## Abstract

Non-ischemic priapism (NiP) is painless partial tumescence caused by genital trauma and the formation of intracorporal arterio-venous fistula. This is a retrospective study of 25 men with NiP and reports the long-term erectile function and colour doppler ultrasound (CDUS) findings after treatment for NiP. Unstimulated CDUS was performed at diagnosis, 1 week and at last follow-up after treatment. CDUS traces were analysed: peak systolic velocity (PSV), end diastolic velocity (EDV), resistive index (RI) and mean velocity (MV) were calculated. Erectile function was assessed using the IIEF-EF questionnaire. At the last follow-up (median 24 months), 16 men had normal erectile function (64%): median IIEF-EF score 29 (IQR 28.5–30; σ^2^ 2.78) and nine had erectile dysfunction (36%): median IIEF-EF score 17 (IQR 14–22; σ^2^ 33.6). MV and EDV were statistically higher in those patients with erectile dysfunction at last follow-up compared to patients with normal erectile function: median MV 5.3 cm/s (IQR 2.4–10.5 cm/s; σ^2^ 34) vs 2.95 cm/s (IQR 1.03-3.95; σ^2^ 3.4) *p* < 0.002 and median EDV 4.0 cm/s (IQR 1.5–8.0; σ^2^ 14.7) vs 0 cm/s (IQR 0–1.75; σ^2^ 2.21) *p* < 0.004. Erectile dysfunction was observed in 36% of men treated for NiP and was associated with abnormal low resistance resting CDUS waveforms. Further investigation for persistent arteriovenous fistulation should be considered in these patients.

## Introduction

Priapism is a prolonged erection that persists more than 4 h in the absence of sexual stimulation [1]. It is a rare urological emergency with an incidence in the general population <1 per 10,000 person years [2, 3]. There are generally 3 recognised subtypes: ischemic, non-ischemic and stuttering priapism. Non-ischemic priapism (NiP) also termed high-flow priapism, is the least common, representing approximately 5% of cases. It is most frequently caused by blunt perineal or iatrogenic injury to the corpora cavernosa resulting in the formation of intracorporal arterio-venous fistula(e) (AVF) typically between the helicine arteries and the sinusoidal veins [4]. AVFs can also complicate corpus cavernosal aspiration and injection in the treatment of ischemic priapism [5].

NiP is diagnosed by corporal blood gas readings demonstrating arterialised oxygen tensions and colour doppler ultrasound (CDUS), which demonstrates a characteristic high flow, low resistance waveform in the cavernosal arteries and turbulent ‘ying-yang’ appearance of the AVF [4, 6].

Conservative management with perineal compression and ice pack application is the initial treatment [1, 7, 8]. Catheter angiography and embolization can be considered in refractory cases and failing this surgical ligation of the cavernosal artery may be required [1]. Erectile dysfunction (ED) can complicate patients treated for NiP via several different mechanisms. These include arterial insufficiency from embolization or surgical ligation [9], veno-occlusive disease [10] and cavernosal fibrosis [8, 11]. There remains no definite guideline on the optimal timing and method of embolization of NiP. Subselective delivery of temporary absorbable embolization agents is thought to reduce the risk of arterial insufficiency by allowing recanalization of the main cavernosal artery after the AVF has thrombosed [9, 12, 13]. Follow-up with CDUS at 1 to 2 weeks after treatment is recommended to confirm successful AVF occlusion [14]. There are few longitudinal studies that provide objective evaluation of CDUS after resolution of NiP [10, 15].

This retrospective study of a cohort of men treated for NiP reports the unstimulated CDUS findings and clinical outcomes at diagnosis, immediately after successful treatment and at late follow up. The objective of this study was to compare the CDUS waveform parameters between patients with different clinical outcomes after treatment for NiP and to identify potential causes of ED.

## Materials and methods

All cases of NiP from a single tertiary andrology centre were retrospectively identified from electronic hospital records between January 2007 and December 2021. Diagnosis of NiP was made following a relevant clinical history of preceding trauma or iatrogenic injury to the corpora and with a corporal blood gas aspiration showing bright red blood and raised oxygen tension (pO2 > 90 mm Hg, pCO2 < 40 mm Hg, pH 7.40) [16]. Patients with a AVF caused by corporal decompression of ischemic priapism were not included in this study. The study sample size was a convenience sample due to the low incidence of NiP.

Unstimulated CDUS was conducted by a consultant uroradiologist within 48 h of presentation. The imaging diagnosis of NiP was made by presence of intracorporal AVF(s) and characteristic low resistance waveforms (low resistive index (RI), high peak systolic (PSV) typically >50 cm/s and end diastolic velocities (EDV) typically >20 cm/s and mean velocity (MV) > 6.5 cm/s in the affected cavernosal artery(s) [17, 18]. Patients were counseled on the treatment options, and a conservative approach with compression and ice pack application was attempted for up to 2 weeks. If unsuccessful, catheter angiography and embolization was performed within 1 month of initial presentation. Inclusion and exclusion criteria are specified in Table 1.

**Table 1:** Inclusion and exclusion criteria for this study.

Inclusion
Diagnosis of acute NiP on blood gas
No previous active treatment for NiP (surgery or embolization)
Normal premorbid erectile (reported function at presentation)
Digital CDUS waveform traces at: diagnosis, early follow-up at 1weeks and late follow-up at 4–24 months after resolution of priapism Digital intraprocedural fluoroscopic imaging
Outpatient andrology and interventional radiology assessments
Exclusion
Prior surgical or intervention for NiP
No or missing CDUS waveforms data at pre or follow-up scans
Loss to follow-up before 4 months after treatment
NiP – Non ischemic Priapism CDUS – Colour doppler ultrasound

All diagnostic and follow up CDUS scans were carried out without the administration of pharcomological stimulants. A 12–18 MHz linear probe on an Acuson 500 (Siemens, Germany) was used. Waveforms were measured in the cavernosal arteries at the most proximal point in the crura; both left and right (and any accessory cavernosal arteries) were sampled. Measurement was carried out over at least three cardiac cycles according to good sonographic practice and in all cases, angle correction was applied [19]. In cases of unilateral AVF, the trace from the cavernosal artery in the affected side was used. In cases of bilateral AVFs, the side with the highest velocity value was used for analysis.

### CDUS protocol and analysis

CDUS waveforms were analysed and Peak systolic velocity (PSV), End Diastolic Velocity (EDV) and Resistive Index (RI) were measured from the velocity trace and Mean Velocity (MV) was calculated. MV is calculated by integrating the velocity trace with respect to time and describes the velocity of blood in an artery throughout the cardiac cycle and therefore combines both the PSV and EDV values in one value [18]. MV was calculated by transferring waveforms to a 1 mm square digital grid and calculated using the equation below:

$$\begin{array}{l}{{{{{{{\mathrm{MV}}}}}}}} = \left( {{{{{{{{\mathrm{Integral}}}}}}}}\,{{{{{{{\mathrm{above}}}}}}}}\,{{{{{{{\mathrm{the}}}}}}}}\,{{{{{{{\mathrm{baseline}}}}}}}} - {{{{{{{\mathrm{integral}}}}}}}}\,{{{{{{{\mathrm{below}}}}}}}}\,{{{{{{{\mathrm{the}}}}}}}}\,{{{{{{{\mathrm{baseline}}}}}}}}\,{{{{{{{\mathrm{in}}}}}}}}\,{{{{{{{\mathrm{mm}}}}}}}}2} \right)/\\ \left( {{{{{{{{\mathrm{length}}}}}}}}\,{{{{{{{\mathrm{cardiac}}}}}}}}\,{{{{{{{\mathrm{cycle}}}}}}}}\,{{{{{{{\mathrm{in}}}}}}}}\,{{{{{{{\mathrm{mm}}}}}}}}} \right) \times ({{{{{{{\mathrm{number}}}}}}}}\,{{{{{{{\mathrm{of}}}}}}}}\,{{{{{{{\mathrm{mm}}}}}}}}\,{{{{{{{\mathrm{squares}}}}}}}}\,{{{{{{{\mathrm{for}}}}}}}}\,{{{{{{{\mathrm{a}}}}}}}}\,{{{{{{{\mathrm{height}}}}}}}}\,{{{{{{{\mathrm{of}}}}}}}}\,100{{{{{{{\mathrm{cm/s}}}}}}}})\end{array}$$

### Angiography technique

Informed written consent was gained from all patients. Angiography was carried out under local anaesthetic via unilateral common femoral or radial artery access. Subselective catheterisation of the anterior trunk of the internal iliac artery was performed with 2.7fr microcatheter (Progreat®, Terumo, Japan). Angiography was performed at the distal Internal Pudendal Artery and the vessels supplying the AVF identified. Nitroglycerin aliquots of 100–200 micrograms were used to prevent arterial spasm. Embolization was performed until no further contrast blush was observed and thrombosis of the AVF was observed on intraprocedural ultrasound.

### Follow-up

Clinical success was defined as a flaccid penis at rest. Examination and follow-up CDUS examinations without pharmacological stimulation were carried out at 1 week, 1 month, and at last follow-up at a range of 4–24 months after return to flaccid state. If a recurrent AVF was detected on follow-up CDUS, further embolization attempts were made until the patient returned to a flaccid state. The follow-up CDUS after the final treatment were then included in the study. Current guidelines on priapism do not explicitly define a follow up period of NiP but in this institution’s experience such patients benefit from a close regular follow up with interventional radiology and andrology for at least 12 months after NiP occurred.

A resting flaccid penis CDUS trace was considered abnormal when a low resistance waveform pattern was detected, defined by: EDV above 5 cm/s, MV above 4 cm/s in the affected cavernosal artery [18, 20]. A normal PSV in the normal resting state is reported as between 10 to 15.9 cm/s (+−5.9 cm/s) [20–22]. In this study, we considered a persistently raised EDV as the primary hallmark of an abnormal CDUS waveform and subsequently evaluated the PSV and whether this had reduced from diagnosis.

Erectile function (EF) was tested by 30-point International Index of Erectile function Erectile function domain A questions (IIEF-EF) and patient subjective reports of EF in andrology clinic. ED was diagnosed if a patient scored less or equal to 25 on the IIEF-EF questionnaire [23]. In selected cases of ED post resolution of NiP, performing a stimulated CDUS was considered subsequent to an unstimulated CDUS in the absence of recurrent priapic state or ongoing AVF.

### Statistical tests

Descriptive statistics and data tested for normal distribution using quantile-quantile plots. Non-parametric tests (Mann–Whitney *U* test) were used to compare continuous variables: CDUS variables, duration of NiP and patient ages. Binary logistic regression analyses were used to ascertain the effects of specific clinical and technical variables including number of AVFs, number of treatment episodes required, embolization agent used had on the likelihood of having ED at last follow-up. Receiver Operating Characteristic curve calculations were made to test the sensitivity and false positive rate of CDUS variables to predict ED. A *P* value less than 5% was considered statistically significant. SPSS® statistical package version 22 (IBM Corp, Armonk, NY, USA) was used for statistical analysis.

### Ethical consideration

After discussion with the local research department, formal ethical committee approval of this retrospective audit of anonymized patient data was not considered necessary.

## Results

A total of 40 consecutive cases of NiP were treated. Twenty-five met the inclusion criteria; 15 were excluded due to inadequate or unavailable pretreatment CDUS traces and/or incomplete follow-up CDUS. The median age was 28 year old (IQR 19–44; σ^2^ 168). All patients reported normal EF before the injury and with no reported comorbidities including diabetes and cardiovascular disease. Perineal injuries were sustained whilst skateboarding (4 cases), cycling (10 cases), other physical activity (10 cases) and iatrogenic injury during routine urethroscopy (1 case).

### Treatment

**Fig. 1:**
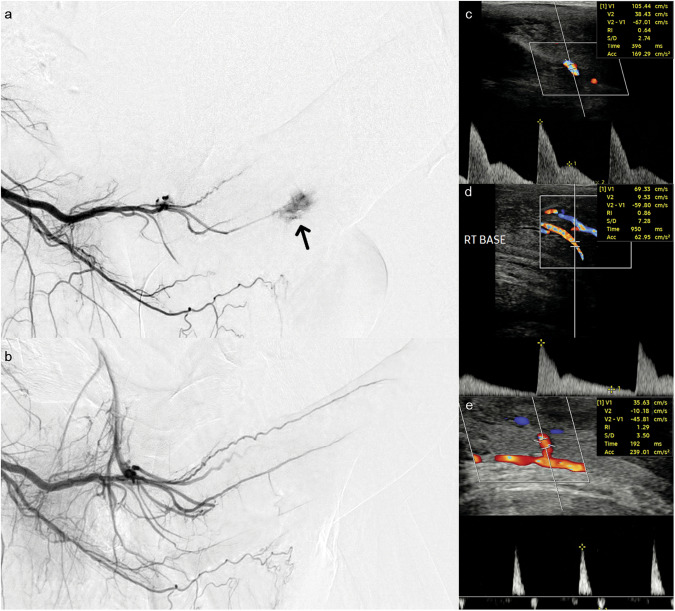
Selected angiographic and ultrasound images from a case of non-ischemic priapism. **a** Angiogram from the distal internal pudendal artery showing AVF (black arrow) arising from a the right cavernosal artery. **b** Post embolization angiogram showing resolution of the AVF and normal filling of the penile artery and branches. **c** Initial diagnostic CDUS trace showing a classical high flow low resistance waveform of NiP (PSV 105 cm/s, EDV 38 cm/s, RI 0.64). **d** Follow up CDUS at 1 week. The patient had a flaccid penis. The waveform is abnormal with the persistent forward flow in diastole (but much improved compared to the initial diagnostic scan (PSV 69 cm/s, EDV 9.5 cm/s, RI 0.86). **e** Subsequent CDUS at 3 months a low flow, high resistance waveform (PSV 39 cm/s, EDV -10 cm/s, RI 1.29). AVF arteriovenous fistula, NiP non-ischemic priapism, CDUS colour doppler ultrasound, PSV peak systolic velocity, EDV end diastolic velocity, RI resistive index.

Each case was treated conservatively with perineal compression over the site of fistula. Three cases resolved with conservative treatment. Twenty-two cases underwent catheter angiography and embolization at a median time of 23 days (IQR 13–92) after onset of NiP. Supplying branches were selected and embolized with 0.5–1 ml of gelfoam slurry, or thrombin. Periprocedural ultrasound was used to confirm thrombosis of the AVF. In 13 cases, 2 mm microcoils (Hilal - Cook) were used to sacrifice a bleeding helicine, but not placed in the main cavernosal artery. Contralateral distal penile angiograms were performed to exclude collateral supply. See Fig. 1. Recurrence of NiP with recanalization of the AVF was detected in 10 cases (40%) at the 1 week follow-up scan, further embolization was then performed at a median of 44 days (IQR 6–55; σ^2^ 223) after initial treatment. 3 patients (12%) required a third embolization to achieve detumescence. All patients had returned to a flaccid state by a median time of 32 days (IQR 19–121; σ^2^ 30637). There were no recorded complications according to the Clavien-Dindo Classification.

### Erectile function

All patients completed the IIEF-EF at last follow-up visit (median 24 months IQR 8–61; σ^2^ 1753). Sixteen patients reported normal spontaneous EF with IIEF-EF scores >25 (median 29; IQR 28.5–30; σ^2^ 2.78). Nine patients had ED with median IIEF-EF of 17 (IQR 14–22; σ^2^ 33.6): in five cases, these had severe ED with a median recorded IIEF-EF of 10 (IQR 10–15; σ^2^ 14.6) and required pharmacological adjuncts to achieve an erection (Cialis 3, Tadanafil 1 and Sildenafil 1); in four cases, men had moderate ED and complained of some reduction in rigidity and occasional early loss of erection hardness (median score 21 IQR 20–22.5; σ^2^ 9.2). The sample size was insufficient to test for significant differences between patients with moderate and severe ED groups. See Table 2.

**Table 2:** Demographics, aetiology of NiP, treatments and outcomes.

	Normal EF	ED
Number of patients	16	9
Age (median and IQR) *p = 0.3 Mann–Whitney *U* test	21 (19.5–40)	27 (22–36.5)
Aetiology		
Fall astride	16	8
Iatrogenic	0	1
Number of AVFs: *p = 0.351 Binomial Logistic Regression		
Unilateral AVF	14	7
Bilateral AVF	2	2
Duration of NiP/days from injury to treatment (median and IQR) *p = 0.18 Mann–Whitney *U* test	28 (16.5–97)	92 (27–121)
Number of Treatment(s): *p = 0.35 Binomial Logistic Regression		
Conservative management	3	0
1x embolization	7	5
2x embolization	4	3
3x embolization	2	1
Embolic type: *p = 0.114 Binomial Logistic Regression		
Gelfoam	1	2
Thrombin	4	0
Thrombin/Gelfoam + Microcoils	8	7
IIEF-EF score at last clinical follow up (median and IQR) *p < 0.0002 Mann–Whitney *U* test	29 (2)	17 (8)

*AVF* arteriovenous fistula, *ED* Erectile dysfunction, *EF* Erectile function, *IIEF-EF* International Index of Erectile Function-Erectile function score, *IQR* interquartile range.

### CDUS parameters

The CDUS waveforms at diagnosis were a high-flow, low-resistance pattern in all cases with median PSV 100 cm/s (IQR 73–160; σ^2^ 3909), median EDV 30 cm/s (IQR 15–45; σ^2^ 409), median MV 45 cm/s (IQR 25–66; σ^2^1127) and median RI 0.69 (IQR 0.57–0.86: σ^2^ 0.02).

After resolution of NiP, there was an immediate reduction in the flow parameters in all patients at 1 week follow-up CDUS (median PSV 25 cm/s IQR 17–35.5; σ^2^ 771, median EDV 5 cm/s IQR 1.75–11; σ^2^ 166, median MV 7 cm/s IQR 3.65–11.65; σ^2^ 54 and median RI 0.80 IQR 0.57–0.81; σ^2^ 0.045). However, in 16 patients (64%) a dampened but persistently low resistance waveform was observed (persisting EDV > 5 cm/s and raised MV > 4 cm/s). This included 10 of the 16 patients who eventually returned to normal EF by late-follow up and 6 of the 9 who had ED at late-follow up. The early follow-up CDUS values in these 16 patients were: median PSV 27.5 cm/s (IQR 23.5–47; σ^2^ 1020), median EDV 10 cm/s (IQR 5.5–11.5; σ^2^ 209), median MV 10 cm/s (IQR 6–16.8; σ^2^ 60.5) and median RI 0.7 (IQR 0.6–0.81; σ^2^ 0.02) compared to the 9 patients with normalised CDUS with median PSV 17 cm/s (IQR11.5–22.5; σ^2^ 107), median EDV 0 cm/s (IQR −1–2.5; σ^2^ 4.43), median MV 3.7 cm/s (IQR2.5–5.1; σ^2^ 3.7) and median RI 1.0 (0.82–1.07; σ^2^ 0.04).

**Fig. 2:**
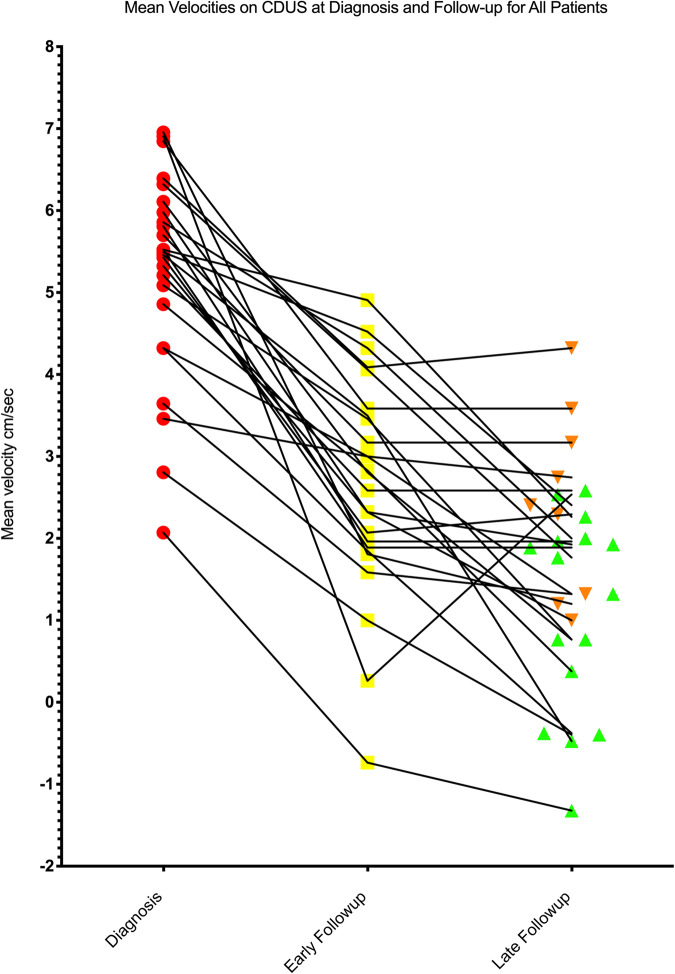
Scatter plot showing the mean velocity in the affected cavernosal artery at diagnosis, early (1 week) and late (3–12 months) follow-up in all patients. Those with erectile dysfunction at the last follow up represented by down-facing triangles and those with normal erectile function at the last follow-up are represented by up-facing triangles.

A further measurable reduction in flow parameters was observed at late follow-up CDUS (at a median of 20 months IQR 6–58; σ^2^1669). All patient data showed a median PSV 23 cm/s (IQR 16.2–29.5; σ^2^115), median EDV 0 cm/s (IQR 0–3.75; σ^2^ 10.4), median MV 3.7 cm/s (IQR 1.7–5.5; σ^2^ 18) and median RI 1.0 (IQR 0.9–1; σ^2^ 0.018). In 10/16 patients with a persistent low resistance waveform identified at early follow-up, CDUS waveforms had returned to an expected normal baseline values by late-follow up with median PSV 20 cm/s (IQR 12.5–31; σ^2^ 228), median EDV 0 cm/s (IQR 0–2; σ^2^ 1.44), median MV 3.4 (IQR 1.2–5.0; σ^2^ 3.82) and median RI 1.0 (IQR 0.9–1.0; σ^2^ 0.0025). At late follow-up in 6 patients (1/16 patients with normal EF and 5/9 with ED) there was a persistent low resistance waveform with median PSV 28 cm/s (IQR 26–30; σ^2^ 10.2), median EDV 5.5 cm/s (IQR 3.8–10; σ^2^ 14.35), median MV 7.85 cm/s (IQR 2.5–12; σ^2^ 40.8) and median RI 0.83 (IQR 0.7–1.0; σ^2^ 0.051). No evidence of a recurrence of the primary AVF on CDUS or clinical recurrence of NiP was observed. See Fig. 2, in 6 of the 9 patients with ED at late follow up, in whom no residual priapism or AVF was identified on unstimulated CDUS, a subsequent stimulated CDUS was performed with 10–20 mcg of intracorporal Alprostadil injection. In all 6 cases, a response to pharmacological stimulation was observed with a minimum threshold of PSV 25 cm/s met (median 43; IQR 29–68.5; σ^2^ 390).

### Comparison of CDUS parameters between normal EF and ED groups

A statistically significant greater MV (median 5.3 cm/s IQR 2.4–10.5, σ^2^ 34 vs 2.95 cm/s IQR 1.03–3.95, σ^2^ 3.4; *p* < 0.002) and EDV (median 4 cm/s IQR 1.5–8.0, σ^2^ 14.7 vs 0 cm/s IQR 0–1.75, σ^2^ 2.21; *p* < 0.004) was measured in patients with ED compared to those with normal EF at late follow-up CDUS (Mann–Whitney *U* test). No statistically significant difference in PSV (median 27 cm/s IQR 19.75–32, σ^2^ 174 vs 20 cm/s IQR 15–26, σ^2^ 71.2; *p* = 0.118) or RI (median 0.9 IQR 0.75–1.0, σ^2^ 0.04 vs 1.0 IQR 0.9–1, σ^2^ 0.005; *p* = 0.258) was seen between these outcomes groups. The CDUS parameters at diagnosis and early CDUS follow-up between ED and normal EF groups did not show any significant difference (see Table 3). No significant difference was seen between the age of patients, median 27 years (IQR 22–36.5; σ^2^ 188) vs 21 years (IQR 19.5–40; σ^2^ 167) (*p* = 0.30) or duration of NiP, median 92 days (IQR27–121; σ^2^ 7508) vs 28 days (IQR 16.5–97; σ^2^ 72079) (*p* = 0.18) between groups. A receiver operating characteristic (ROC) curve was plotted using the MV data at the time of last-follow up CDUS to assess the diagnostic accuracy of identifying patients with ED at last follow-up. The area under the curve was 0.79 (standard error 0.098; *p* < 0.02; 95% Confidence Interval 0.59–0.98). A cut off MV value of 4.4 cm/s (sensitivity of 66.7% and false positive rate of 18.8%) predicted presence of ED at last follow-up.

**Table 3:** CDUS parameters summarised at diagnosis, early and late follow up after treatment.

	CDUS At Diagnosis (median and IQR)			CDUS Early follow-up 1 week (median and IQR)			CDUS Late follow-up 4–24 months (median and IQR)		
	All	Normal EF	ED	All	Normal EF	ED	All	Normal EF	ED
PSV cm/s	100 (73–160)	108.5 (74.5–148)	85 (72.5–224)	25 (17–35.5)	24.5 (15.8–35)	25.5 (19.5–35.5)	23 (16.2–29.5)	20 (15–26)	27 (19.75–32)
EDV cm/s	30 (15–45)	21.5 (12.5–45)	33 (20.5–40.5)	5 (1.75–11)	4 (0.75–10.5)	6.1 (3.5–11)	0 (0–3.75)	0 (0–1.75)	4 (1.5–8.0)
RI	0.69 (0.57–0.86)	0.68 (0.56–0.88)	0.7 (0.61–0.83)	0.8 (0.57–0.81)	0.82 (0.6–0.96)	0.78 (0.52–0.85)	1 (0.9–1)	1.0 (0.9–1.0)	0.9 (0.75–1.0)
MV cm/s	45 (25–66)	45 (24.5–60.5)	45 (30–99.5)	7 (3.65–11.65	5.5 (3.3–11.15)	8 (4.6–14.5)	3.7 (1.7–5.5)	2.95 (1.03–3.95)	5.3 (2.4–10.5)
		Mann–Whitney *U* test *p* = 0.97 PSV *p* = 0.39 EDV *p* = 0.75 RI *p* = 0.53 MV			Mann–Whitney *U* test *p* = 0.84 PSV *p* = 0.43 EDV *p* = 0.23 RI *p* = 0.23 MV		Friedman test for repeated variables: *X*^2^_*r*_ statistic was 41.42 (2, *N* = 25) *p* < 0.0001	Mann–Whitney *U* test *p* = 0.118 PSV *p < 0.004 EDV *p* = 0.258 RI *p < 0.002 MV	

*CDUS* Colour Doppler Ultrasound, *EF* Erectile function, *ED* Erectile dysfunction, *PSV* Peak Systolic Velocity, *EDV* End Diastolic Velocity, *RI* Resistive Index, *MV* Mean velocity, *IQR* 1–3rd Interquartile range.

Binary logistic regression analysis did not show a significant difference in the number of AVFs (median 1 for both groups) *p* = 0.351; type of embolization agent either coils vs temporary embolic (*X*^2^ = 2.4934 *p* = 0.114) or number of treatments (median 2 treatments vs 1 treatment) *p* = 0.35 between patients with ED or normal EF at late follow-up (for details of embolic agent please see Table 2).

## Discussion

This study presents long term clinical and imaging follow up in a large group of patients with NiP identified in a single centre. The principal findings of this study are 36% of men have ED at late clinical follow up (median 24 months IQR 8–61; σ^2^ 1753). Unstimulated CDUS demonstrates a marked change in the resting penile hemodynamic state before and after treatment of NiP: at diagnosis there is a low resistance, high flow waveform (high PSV, EDV and MV). After treatment there is rapid reduction in flow (reduced PSV, EDV and MV) and increased resistance (RI), which continues to improve over several months and returns to a normal baseline in the majority of cases.

NiP develops several hours after the initial injury, thought to be due to an initial period of vascular spasm with subsequent AVF formation during normal physiological erections [24]. CDUS is the most sensitive and specific tool to locate AVFs and waveforms are characteristic, with a low resistance and high flow. MV can reliably characterise the altered hemodynamics in NiP and can be used to differentiate between ischemic and non-ischemic priapism [18]. In the normal flaccid penis the resting CDUS demonstrates high resistance waveforms with a low PSV and zero or reversed EDV. The reported normal EDV in the flaccid state is 0.34 + −1.5 cm/s [20] whilst a normal resting PSV is reported over a broader range between <10 cm/s +−9 cm/s to 15.9 + −5.9 cm/s by different studies [20–22]. This likely reflects the heterogeneity in resting PSV values which are dependent on several factors including office temperature and patient anxiety.

Embolization for NiP is performed with superselective catheterisation and blockage of the AVF with temporary embolics (including thrombin and gelfoam). Microcoils are useful to occlude small helicine vessels whilst preserving the main cavernosal arterial supply. Embolization causes thrombosis of the AVF [10], resulting in a return to normal high resistance waveform with very low or reversed EDV and reduction in PSV. CDUS waveforms can rapidly normalise after successful embolization [15], but persisting low-resistance, high-flow waveform has also been observed, which typically resolve over several weeks [10]. The persistent low resistance waveform is thought to represent hyperemia as part of tissue healing and/or small arterio-venous connections [10] and is indicated by the persist raised EDV and MV. Our study data show a large proportion of patients (64%) followed the latter pattern with rapid reduction in flow after treatment but with a measurable persistent abnormal low resistance and raised MV. Blood flow returned to a normal resting waveform at a median of 20 months (IQR 6–58; σ^2^1669) in the majority of cases (76%).

ED post embolization for NiP is observed in between 8–39% cases of reported series [1, 9, 12, 13, 25, 26]. The mechanism is unknown but veno-occlusive dysfunction, oxygen free radical damage leading to corporal fibrosis and arterial insufficiency are proposed mechanisms [6, 8, 11, 27], whilst arterial insufficiency is less likely with modern subselective embolization techniques [10]. In the 6 patients with ED that underwent a stimulated CDUS, the median PSV was 43 cm/s, which does not support a diagnosis of arterial insufficiency. Incomplete treatment of the AVF is also a known cause of ED despite a state of partial tumescence [6, 25].

Thirty-six percent of this cohort had ED at late follow up. Persistent abnormal low resistance waveforms on unstimulated CDUS were observed more frequently in patients with ED. This was characterised by a significantly raised MV and EDV (cut-off value of MV 4.4 cm/s ROC) with the absence of a defined AVF and recurrence of NiP. This finding suggests that low grade venous shunting may be contributing to ED due to small arterio-venous connections. The management of this poses several challenges to both interventional radiologists and andrologists, as there may no clear target for subselective embolization and non-selective treatment may risk arterial insufficiency. Careful assessment with pharmacologically stimulated CDUS, cavernosography and, in select cases, subselective angiography and judicious further embolization may have a role.

There are several limitations to this study: although all patients meeting our minimum criteria for imaging and clinical follow up were included, many were excluded, potentially biasing the group and reducing the size of the study population to a relatively small convenience sample. This study remains one of the largest series of NiP with long-term follow up. Due to the emergent nature of NiP it was not possible to formally test premorbid EF. Individuals were excluded if they did not report subjective premorbid normal EF. CDUS is a technically challenging technique with intraobserver variability and not all patients with ED proceeded to a pharmacologically stimulated CDUS as part of their ED workup. Those men with ED did not undergo further investigation for ED with for instance with cavernosography or nocturnal penile tumescence and rigidity tests.

Thirty-six percent of men treated for NiP had ED at last follow up. Follow-up CDUS commonly shows a persisting low resistance waveform 1 week after successful treatment of NiP. In the majority of patients this resolves, but if it persists, a raised resting MV and EDV in the cavernosal arteries can be associated with ED and suggests ongoing arterio-venous connections. This finding can inform decisions on management including investigation with stimulated CDUS, cavernosography and consideration of further angiography and embolization.

## Data Availability

Data are available from the corresponding author on reasonable request.
