# Correction: Therapeutic outcomes and analysis of Doppler findings in 25 patients with non-ischemic priapism

**DOI:** 10.1038/s41443-024-00936-0

**Published:** 2024-06-21

**Authors:** Conrad von Stempel, Rohaan Shahzad, Miles Walkden, Fabio Castiglione, Asif Muneer, David Ralph, Alex Kirkham

**Affiliations:** 1 Department of Radiology, University College London Hospitals, London, UK; 2 Division of Surgery and Interventional Science, University College London, London, UK; 3 Department of Urology, King’s College Hospital NHS Foundation Trust, London, UK; 4 Department of Urology, University College London Hospitals, London, UK

**Keywords:** Erectile dysfunction, Diagnosis

Correction to: *International Journal of Impotence Research* 10.1038/s41443-023-00719-z, published online 13 June 2023

In this article, the author’s name “Shahzad” was incorrectly written as “Shazhad”.

